# A causal moderated mediation analysis of maternal prenatal bonding and daily infant engagement on postpartum bonding

**DOI:** 10.3389/fped.2026.1908967

**Published:** 2026-08-11

**Authors:** Caitlin Dressler, Stacy Tiemeyer, Karina M. Shreffler

**Affiliations:** Fran and Earl Ziegler College of Nursing, University of Oklahoma Health Campus, Oklahoma City, OK, United States

**Keywords:** bonding, caregiving, maternal, PAI, PBQ, postpartum, prenatal

## Abstract

**Background:**

The early maternal-infant bond has profound impacts for infant development. Prior research indicates that higher self-reported maternal-fetal bonding predicts greater postpartum bonding, but additional research is needed on the mechanisms that may promote the early maternal-infant relationship. We explored daily engagement in caregiving behaviors as a potential non-pharmacological intervention to promote maternal and infant health.

**Methods:**

This study utilizes a diverse and predominately low-income sample of 114 mothers of infants who were recruited at their first prenatal visit in 2017–2018 and followed through at least six months post-birth. We conducted a causal mediation analysis to examine whether daily engagement at two months postpartum functions as a mediator in the maternal prenatal and postpartum bonding relationship, and additionally tested whether this mediation was moderated by maternal prenatal bonding levels for postpartum bonding.

**Results:**

Maternal prenatal bonding was associated with daily engagement (*b* = 0.03, *SE* = 0.01, *p* < .001) and postpartum bonding (*b* = 0.28, *SE* = 0.08, *p* = .001), controlling for covariates. We also found that daily engagement at two months postpartum was associated with bonding at six months postpartum (*b* = 3.88, *SE* = .95, *p* < .001). Causal mediation analyses indicated a significant indirect effect of prenatal bonding on postpartum bonding through daily engagement among mothers with lower levels of prenatal bonding. There was also evidence of moderation; daily engagement was a stronger predictor of postpartum bonding among mothers with lower prenatal bonding, whereas mothers with higher prenatal bonding showed consistently strong bonding regardless of engagement level (adjusted R^2^ = .378).

**Conclusions:**

The results of this study indicate that maternal engagement activities may help to facilitate postpartum bonding and may explain the association between prenatal and postpartum bonding. Additionally, engaging in more caregiving activities may strengthen postpartum bonding, particularly among those with lower prenatal bonding. These findings suggest promoting maternal engagement in caregiving behaviors may promote a stronger postpartum bond.

## 1 Introduction

The early maternal-infant relationship is critical for a child's future (1), as it has profound implications for later life social, cognitive, behavioral, and physical development (2). Primary caregivers, usually mothers, provide their infants with needed safety, security, and comfort, and a special bond, or attachment, grows between them (3). When the bond is strong, it is characterized by mothers who are highly sensitive and responsive to their infants' cues, and by infants who seek comfort from their caregivers during times of stress (4).

The early maternal-infant bonding relationship begins to form during pregnancy (5) and continues to evolve throughout the pregnancy and into the postpartum period (6). Prenatally, the mother-infant bond is often referred to as maternal-fetal attachment (MFA) and is influenced by contextual and environmental factors such as parity, level of social support, and maternal physical and mental health and well-being (7). MFA refers to the extent to which a pregnant woman is emotionally connected to and demonstrates behaviors of connection and interaction with the unborn fetus (5). Feelings of prenatal bonding can occur prior to a pregnancy, as women are able to conceptualize a future child (8), but MFA can be quite low at the beginning of a pregnancy, particularly when it was unexpected and unwanted (9). MFA increases through pregnancy as the mother increasingly feels movement and as the mother has more idealizations and expectations about the future newborn (10). Following delivery, MFA transitions into the maternal-infant bond, and this relationship continues to develop through proximity and the emotional connection towards the infant that was established during pregnancy (6). Although bonding increases throughout pregnancy and into the postpartum period, levels of MFA vary considerably and predict subsequent postpartum bonding (9, 11–14).

The maternal-infant bond facilitates mothers' affection for their infants and the desire to care for their needs, and it affects mothers' confidence to be able to provide for those needs (15). The emotional connection felt by mothers and the behavior that they then exhibit through caring for their children is a vital prerequisite for infants to feel secure, protected, and to develop autonomy (16). This establishes the attachment infants have for their mothers and enables infants to feel supported enough to explore and develop social and relational skills that are important for their development (15, 17, 18).

Maternal caregiving behaviors help to facilitate growth of the maternal-infant bonding relationship (2). The maternal caregiving behaviors that facilitate the early mother-infant relationship include the routine activities that a mother may perform to spend time with and care for the newborn, such as bathing and feeding. Caregiving activities are measurable and modifiable behavioral exercises that increase the interaction between a mother and her infant. For example, subtle and routine actions such as kissing, cuddling, and soothing the infant have been expressed as positive indicators that a bond is present or being established (15, 19, 20). The increased proximity that is facilitated through a mother's routine engagement with her infant has been identified as a key mechanism for promoting the postpartum bond (20).

Because the maternal-infant bond is vital for the health and well-being of the mother and for the infant to achieve optimum development (2, 18, 21, 22), identifying factors that may contribute to the development of this bond is essential for interventions designed to promote the maternal-infant relationship (17, 20, 23). Prior research has focused on how specific maternal behaviors may influence bonding including holding skin-to-skin (24), rooming in (25, 26), and singing nursery rhymes (22, 27, 28). However, this is the first study to our knowledge to focus on self-reported engagement in eight daily caregiving activities (e.g., singing nursery rhymes, changing diapers, feeding, bathing, etc.) to determine if maternal caregiving engagement in the early postpartum period serves as a mediator between MFA and the maternal-infant bonding relationship at six months postpartum. We hypothesize that higher levels of MFA will be associated with higher levels of engagement in caregiving activities in the early postpartum period, and that in turn, caregiving engagement will be associated with subsequent postpartum bonding. Additionally, we explore whether the mediating impact of caregiving activities is moderated by MFA. Exploring the mechanisms driving the translation of MFA to a strong maternal-infant postpartum bonding relationship can inform interventions and clinical practice improvements in maternal and infant care.

## 2 Methods

### 2.1 Data source and study population

Participants were recruited in 2017–2018 from two university-affiliated women's health clinics in the northeast region of Oklahoma at the time of their first prenatal appointment and were followed through six months postpartum. Women were excluded from the study if they were more than 16 weeks gestation, younger than 15 or older than 45, not planning to continue their pregnancies, not planning to raise their children as a primary caregiver, or if they were unable to speak and read English. Enrolled participants completed online self-report questionnaires across nine waves during pregnancy, the postpartum period, and early motherhood through two years after delivery. The analytic sample for this study included 114 predominately low-income and racially diverse mother-infant dyads who completed the second trimester survey, the two-month post birth assessment, and the six-month postpartum survey. Participants self-identified as non-Hispanic White (42%), African American (28%), Native American (18%), and Hispanic (12%) women ranging in age between 16 and 38 years old, with an average age of 26. More than half (55%) of the women were married or cohabiting, and most experienced economic hardship (e.g., experiencing at least one economic strain) in the past year. The study was approved by the authors' Institutional Review Board.

### 2.2 Measures

Postpartum bonding was assessed with the *Postpartum Bonding Questionnaire* (PBQ) at six months postpartum (29). The PBQ is a 25-item questionnaire, measuring a range of emotional and behavioral responses toward the infant, including feelings of closeness and affection, irritation or anger, anxiety related to caregiving, and rejection. Mothers rate how often each statement applies to them on a 6-point scale from “never” to “always,” with higher scores coded in this study to indicate higher postpartum bonding. Because the original PBQ was designed to reflect higher scores indicating difficulties in bonding, we reverse coded the appropriate items so that a higher score reflects stronger postpartum bonding. Internal consistency in this sample was *α* = .93.

Maternal-fetal attachment (MFA) was assessed using the *Prenatal Attachment Inventory (PAI)* during the second trimester (30). The 21-item scale assesses multiple aspects of prenatal attachment, including emotional, cognitive and behavioral components of the developing bond between a mother and her baby. Each item describes pregnancy experiences (e.g., feeling love for the baby, imagining the baby's personality, touching the abdomen to interact with the fetus) and is rated on a 4-point Likert scale ranging from “almost always” to “almost never”. Total scores range from 21 to 84, with higher scores indicating stronger prenatal attachment The Cronbach's reliability coefficient of the scale is high in this sample at *α* = .94.

Maternal engagement at two months postpartum was measured using a modified measure from the Child Development Supplement to the Panel Study of Income Dynamics (31). The *Daily Engagement* measure captures the frequency of mothers' interactive behaviors with their infants across a typical week, including singing, talking, reading, and playing. Participants were asked, “In a typical week, how often do you: sing songs or nursery rhymes, read stories, take the baby to visit family, change the baby's diaper, feed or give the baby a bottle, kiss or show physical affection, put the baby down for a nap, or give the baby a bath”. Mothers reported how many days per week they typically performed each behavior (0–7 days), and items were averaged to create an overall index of weekly engagement. These activities were included to broadly capture routine opportunities for mothers to engage with their babies. Higher scores indicate more frequent mother–infant engagement. The index demonstrated good internal consistency in the current sample (Cronbach's *α* = .80).

We conducted a series of sensitivity analyses to assess the potential influence of infant health characteristics on study outcomes. Specifically, we examined whether multiple birth status, low birth weight, prematurity, and NICU admission were associated with maternal engagement or postpartum bonding. Results indicated that prematurity and NICU admission were the infant factors most strongly associated with postpartum bonding; therefore, these variables were included as covariates in the final models.

### 2.3 Statistical analysis

All analyses were conducted in Stata (version 19.5). We computed means, standard deviations, and ranges for all continuous variables, and proportions for categorical variables. Cronbach's alpha was used to assess internal consistency of multi-item scales (Table 1). We used regression modeling to estimate the mediator and outcome paths (Table 2), and causal mediation analysis testing whether daily engagement functions as a mediator, and whether this mediation was moderated by MFA in predicting postpartum bonding. To estimate causal mediation, we used Stata's mediate command. Table 3 summarizes the indirect, direct and total effects estimated in the moderated mediation model. For interpretation of moderated mediation effects, MFA was mean-centered and conditional effects were estimate one standard deviation below (Low MFA −12.47) and one standard deviation above (High MFA +12.47) the mean.

**Table 1:** Descriptive statistic.

Variables	M/%	SD	Min	Max	α
Postpartum Bonding	127.58	11.17	62	135	.93
Maternal-Fetal Attachment	63.75	12.51	36	84	.94
Daily Engagement	5.39	1.01	1.25	7	.80
Premature Birth	19%				
NICU	9%				
Race/Ethnicity					
White	42%				
Black	28%				
Hispanic	12%				
Native American	18%				
Age	25.85	5.53	16	38	
Married/Cohabiting	55%				
Economic Hardship	1.53	1.89	0	6	.91
*N*	114				

Cronbach’s alpha (α) reflects the internal consistency of multi-item scales.

**Table 2:** Multiple regression analysis exploring the associations between maternal-fetal bonding, maternal-infant engagement, and bonding at 6 months postpartum (*N* = 114).

Standard Error (SE)	Outcome=Daily engagement		Outcome=Postpartum bonding					
*p*-value	(1)		(2)		(3)		(4)	
Maternal Fetal Attachment	.03***	(.01)	.28***	(.08)	.17*	(.08)	.15*	(.07)
Daily Engagement					3.88***	(.95)	1.85*	(.93)
MFA × Daily Engagement							−.31***	(.06)
Premature Birth	−.25	(.24)	−5.36*	(2.52)	−4.40	(2.36)	−3.42	(2.11)
NICU	−.45	(.33)	−8.57*	(3.45)	−6.82*	(3.25)	−9.80**	(2.94)
Age	.01	(.02)	−.19	(.19)	−.23	(.18)	−.26	(.16)
Married/Cohab^a^	−.02	(.20)	−3.80	(2.07)	−3.73	(1.93)	−3.60*	(1.72)
Economic Hardship	−.01	(.05)	−.89	(.51)	−.84	(.48)	−.69	(.43)
Race/Ethnicity^b^								
Black	−.39	(.24)	−1.39	(2.47)	.11	(2.33)	.35	(2.07)
Hispanic	−.14	(.31)	−.99	(3.26)	−.45	(3.04)	−2.38	(2.73)
Native American	.20	(.26)	1.86	(2.71)	1.10	(2.53)	−.16	(2.27)
Constant	5.31***	(.53)	137.85***	(5.53)	138.18***	(5.15)	140.37***	(4.60)
*N*	114			114		114		114
adj. R^2^	.115			.212		.315		.458

Standard errors in parentheses.

* *p* < 0.05, ** *p* < 0.01, *** *p* < 0.001.

a Ref=single.

b Ref=white.

**Table 3:** Causal mediation analysis.

Effect	Contrast	*b*	SE	*p*	Lower 95% CI	Upper 95% CI
NIE	−12.47 vs. 0	−1.62	.80	.042*	−3.19	−.06
NIE	+12.47 vs. 0	−0.61	.55	.264	−1.69	.46
NDE	−12.47 vs. 0	−2.00	.85	.019*	−3.67	−.33
NDE	+12.47 vs. 0	2.00	.85	.019*	.33	3.67
TE	−12.47 vs. 0	−3.62	1.09	.001**	−5.76	−1.49
TE	+12.47 vs. 0	1.39	.81	.088	−.21	2.98

MFA and Daily Engagement are centered at the means to test interaction effects. Contrast =  ±1 SD vs. Mean.

* indicates *p* < 0.05, ** indicates *p* < 0.01.

## 3 Results

Overall, respondents reported high levels of postpartum bonding (m = 127.58, SD = 11.17) (see Table 1). Maternal–fetal attachment scores averaged 63.75 (SD = 12.51), indicating that, overall, participants reported moderately high levels of prenatal bonding (Table 1). Daily engagement was the mean number of days per week (1–7) mothers reported performing eight routine caregiving and interaction behaviors; on average, mothers engaged 5.39 days/week (SD = 1.01; range = 1–7), indicating generally high engagement.

Table 2 presents the effect of MFA on daily engagement and PBQ. In Model 1, predicting daily engagement, higher MFA was associated with more frequent weekly engagement behaviors (*b* = 0.03, *SE* = 0.01, *p* < .001) after adjusting for covariates. Models 2–4 predict our outcome of interest, PBQ. As expected, MFA showed a positive association with PBQ scores (*b* = 0.28, *SE* = 0.08, *p* < 0.01). When daily engagement was added to the model, MFA remained a significant predictor of postpartum bonding (PBQ) (*b* = 0.17, *SE* = 0.08, *p* < .05). Daily caregiver engagement at two months postpartum was associated with bonding at six months postpartum (*b* = 3.88, *SE* = .95, *p* < .001), adjusted for covariates. Model 3 increased the explained variance from 21% to 32%. The interaction model (Model 4) revealed a significant MFA × Daily Engagement interaction, indicating that daily engagement was most strongly associated with higher levels of postpartum bonding among mothers who reported lower levels of prenatal bonding, whereas mothers with higher prenatal bonding reported greater postpartum bonding regardless of engagement level. In the fully adjusted model, NICU admission was associated with lower postpartum bonding (*b* = −9.80, *SE* = 2.94, *p* < .01), whereas prematurity was not significantly associated with bonding. The MFA × Daily Engagement interaction remained significant, and the explained variance increased to 46% (adjusted R^2^ = .458).

**Figure 1:**
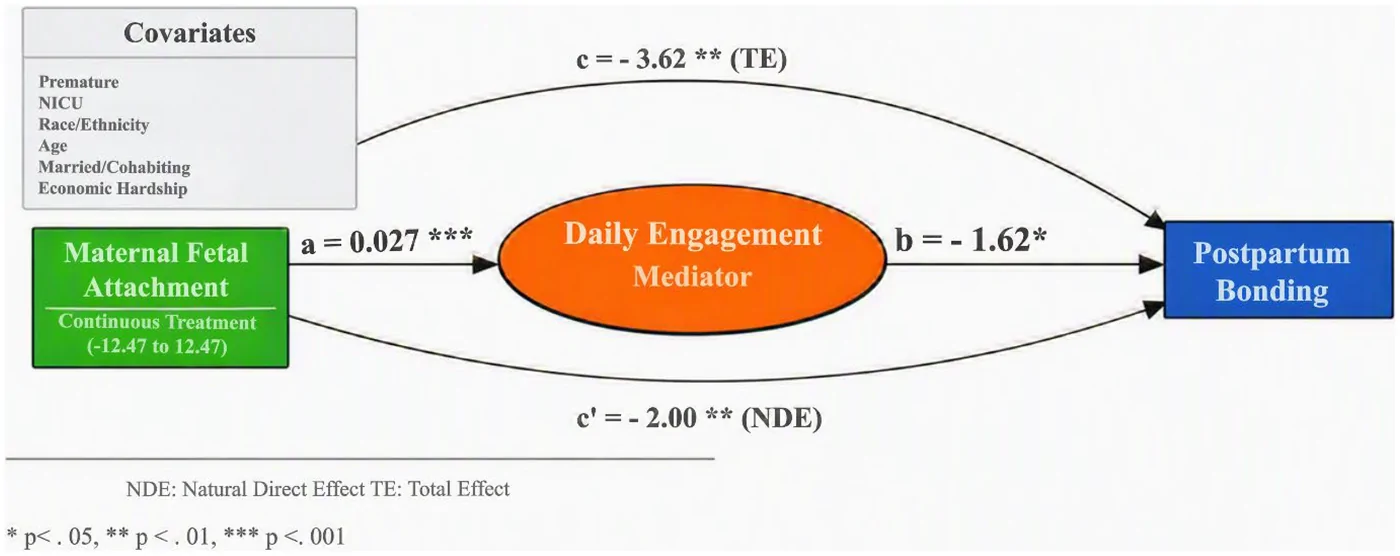
Conceptual moderated mediation model illustrating the indirect effect of maternal fetal attachment (MFA) on postpartum bonding through daily engagement.

Table 3 presents the moderator–mediator interaction, indicating that the effect of daily engagement on postpartum bonding depends on the level of MFA. In this conditional process model, MFA (X) during the 2nd trimester predicted daily engagement (M) at two months postpartum, which in turn influenced bonding (Y) at six months postpartum; however, the strength of the daily engagement → postpartum bonding path varied as a function of MFA, producing different indirect effects at different MFA values. The significant natural indirect effect (NIE) at lower MFA levels (*b* = −1.62, *SE* = 0.80, *p* = .042) indicates that daily engagement partly explains the association between low prenatal bonding and low postpartum bonding, whereas the nonsignificant NIE at higher MFA (*b* = −0.61, *SE* = 0.55, *p* = .264) suggests minimal mediation when prenatal bonding is strong. The natural direct effect (NDE) was also significant at both low and high levels of MFA (|*b*| = 2.00, *SE* = 0.85, *p* = .019), indicating that prenatal bonding remained directly associated with postpartum bonding beyond its indirect pathway through daily engagement. Similarly, the total effect was significant at lower levels of MFA (*b* = −3.62, *SE* = 1.09, *p* = .001), but not at higher levels of MFA (*b* = 1.39, *SE* = 0.81, *p* = .088). Figure 1 presents these results conceptually for the causal moderated mediation model.

**Figure 2:**
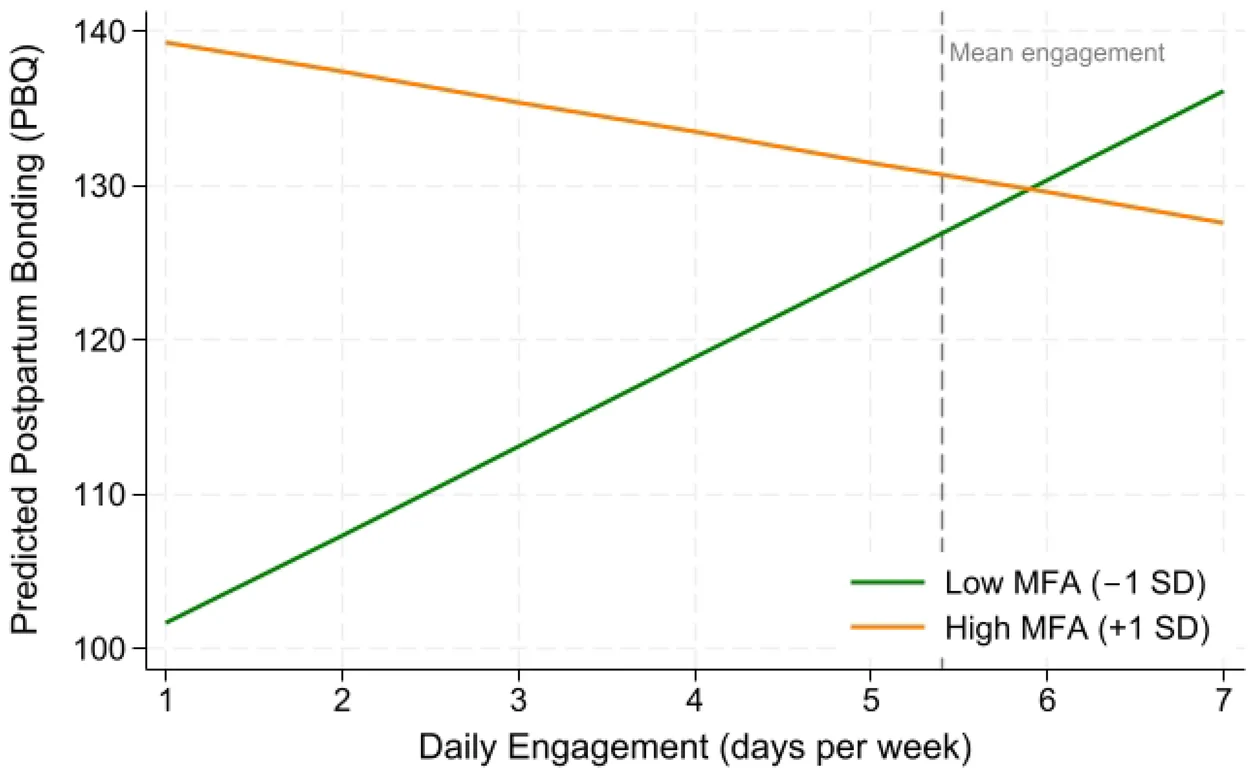
Postpartum bonding by daily engagement at low vs. high maternal-fetal attachment.

Figure 2 shows that the positive association between daily engagement and postpartum bonding is strongest among mothers with lower prenatal bonding (steep green slope), whereas mothers with higher prenatal bonding maintain high postpartum bonding across engagement levels (flat orange slope).

## 4 Discussion

As hypothesized, we found that when mothers report higher levels of attachment during pregnancy, they report more interactive and engaged caregiving behaviors with their infants after birth, which may contribute to a stronger postpartum bond between mothers and infants. Additionally, we found that maternal daily engagement in early caregiving behaviors was associated with the development of the maternal-infant bonding relationship, and that daily engagement matters most for mothers who had reported lower levels of prenatal bonding. This finding provides a non-pharmacological target for intervention and prevention, as providers and childbirth educators can share with mothers the importance of being engaged and active in the daily care of their children and the role that early caregiving behaviors may have on the early bonding relationship with their children. These caregiving behaviors not only meet the needs of their children, but they also strengthen the early bonding relationship, which is critical for infant development (20, 23).

Prior studies focused on identifying modifiable targets for interventions to promote healthy bonding often focus on singular behaviors like skin-to-skin care (24), feeding practices (32), or singing (22, 27, 28). The present study is one of the first to examine how a cluster of caregiver exercises performed daily have the potential to promote the maternal-infant bond—particularly among mothers with lower levels of prenatal bonding. The results of this study suggests that more engagement in daily activities of caregiving is associated with stronger early bonding. These maternal behaviors are associated with proximity and interactions with the infant, which in turn facilitates growth of the mother-infant bond. Providers should be aware of the importance of maternal daily engagement in caregiving activities for bonding, particularly for women who report lower emotional attachment during pregnancy, such as women who have an unintended pregnancy (9). Providers can work with pregnant women and families to increase awareness about the importance of caregiver-infant relationships and the role of regular engagement with the infant as a way to increase feelings of connection and bonding.

### 4.1 Strengths and limitations

These findings should be interpreted considering several strengths and limitations of the study. First, the prospective, longitudinal nature of the study enabled a temporal examination of the development of the maternal-infant bonding relationship across pregnancy and into the postpartum period and mediating factors. Additionally, the study utilized validated bonding measures, strengthening confidence in the reliability of the study findings. There were some limitations to consider as well. All measures in the study were based on self-reports, including daily engagement with caregiving activities. Self-reported behaviors can be biased, particularly when participants perceive a social-acceptability aspect to their responses (33). Survey responses also capture static responses to constructs that can shift over time or based on the participant's mood at the time of the survey (34). Objective measures of bonding or caregiver engagement, such as those captured using observational research, would strengthen confidence in the study findings. Additionally, although the sample was diverse, the small sample size and regional clinical nature of the sample might limit generalizability of our findings. Future research could replicate this study using a representative sample or examine how interventions designed to promote greater maternal engagement affect the early maternal-infant relationship and infant development.

## 5 Conclusion

The results of this study document the role of daily caregiver engagement for the maternal-infant bonding relationship. Daily engagement in caregiving in the early postpartum period was found to mediate the association between prenatal bonding assessed during the second trimester and postpartum bonding assessed at six months post-birth. Moreover, the positive association between daily engagement on postpartum bonding is greater for women who reported lower levels of prenatal bonding. Daily engagement in caregiving activities does not matter as much for bonding among women who reported high prenatal bonding; their self-reported bonding levels are consistently high regardless of their daily engagement. Because the bond between mother and child is critical for optimal infant development, identifying modifiable behaviors such as caregiver engagement that can help promote the early maternal-infant attachment relationship, particularly among those at greatest risk for bonding impairments, is essential for the promotion of maternal and infant health.

## Data Availability

The raw data supporting the conclusions of this article will be made available by the authors, without undue reservation.
